# A Phase II Trial of Perioperative Camrelizumab Plus Neoadjuvant Chemotherapy in Resectable Stage IIB–IIIB Lung Squamous Cell Carcinoma

**DOI:** 10.1002/mco2.70793

**Published:** 2026-06-15

**Authors:** Mingming Hu, Shuku Liu, Yi Han, Feng Wang, Yang Liu, Baohua Lu, Hongxia Li, Yuan Gao, Ying Wang, Wei Yang, Kang Shi, Bo Xiao, Qunhui Wang, Juan Du, Haifeng Lin, Xiaomi Li, Chong Wang, Tongmei Zhang

**Affiliations:** ^1^ Department of Oncology Beijing Chest Hospital, Capital Medical University Beijing China; ^2^ Laboratory For Clinical Medicine Capital Medical University Beijing China; ^3^ Department of Thoracic Surgery Beijing Chest Hospital, Capital Medical University Beijing China; ^4^ Department of Pathology Beijing Chest Hospital, Capital Medical University Beijing China

**Keywords:** camrelizumab, lung squamous cell carcinoma, major pathological response, neoadjuvant immunochemotherapy, perioperative immunotherapy

## Abstract

This phase II trial investigated perioperative camrelizumab plus neoadjuvant chemotherapy for patients with stage IIB–IIIB lung squamous cell carcinoma (LUSC) and explored predictive biomarkers of treatment response. Patients received neoadjuvant camrelizumab (200 mg, Day 1) in combination with nab‐paclitaxel (130 mg/m^2^, Days 1 and 8) and carboplatin (area under the curve = 5, Day 1) every 3 weeks for two cycles, followed by surgery and adjuvant camrelizumab for 1 year. The primary endpoint was major pathological response (MPR) rate. Forty‐five patients were treated, of whom 41 underwent surgery. Twenty‐seven of 45 patients (60.0%) achieved MPR and 20 (44.4%) achieved pathological complete response (pCR). With a median follow‐up of 37.5 months, the 3‐year disease‐free survival and overall survival rates were 65.9% and 68.7%, respectively. Among eight patients with stage IIIB disease, three (37.5%) achieved pCR. Baseline neutrophil percentage and smoking history were associated with MPR. T‐cell receptor (TCR) sequencing revealed that three V‐J gene pairs in TCR beta clones differed between the pCR and non‐pCR groups. This study supports the application of perioperative immunotherapy combined with neoadjuvant chemotherapy in patients with resectable locally advanced LUSC. The identification of blood biomarkers for precise patient selection warrants further in‐depth investigation.

## Graphical abstract

This phase II trial suggests the potential of perioperative camrelizumab plus neoadjuvant chemotherapy in patients with resectable stage IIB‐IIIB lung squamous cell carcinoma. The major pathological response rate (primary endpoint) was 60.0%, and the pathological complete response (pCR) rate was 44.4%. Three V‐J gene pairs in T‐cell receptor beta clones differed between the pCR and non‐pCR groups. Identification of blood biomarkers for patient selection warrants further investigation.

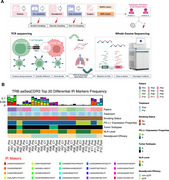

## 1 Introduction

Lung cancer is the most prevalent and lethal malignancy worldwide, with high incidence and mortality rates [1]. Non‐small cell lung cancer (NSCLC) accounts for approximately 85% of all lung cancer cases [2]. In recent years, chemoimmunotherapy has markedly improved the prognosis of patients with advanced NSCLC and has become the standard first‐line treatment. Furthermore, several landmark phase III trials, including CheckMate 816 [3], KEYNOTE‐671 [4], AEGEAN [5], and CheckMate 77T [6], have firmly established the clinical value of chemoimmunotherapy as neoadjuvant or perioperative treatment for resectable NSCLC. Although these trials enrolled both lung squamous cell carcinoma (LUSC) and non‐squamous cell carcinoma patients, LUSC differs substantially from lung adenocarcinoma in terms of molecular features and tumor immune microenvironment.

Previous studies have suggested that patients with LUSC may derive greater benefit from immunotherapy than those with non‐squamous NSCLC, which may be attributed to higher programmed cell death‐ligand 1 (PD‐L1) expression and more abundant immune cell infiltration within the tumor microenvironment of LUSC [7]. However, few clinical trials have exclusively focused on neoadjuvant immunotherapy in this subtype. In addition, only approximately one‐third of patients with NSCLC derive significant clinical benefit from immune checkpoint inhibitors (ICIs). A poor response to neoadjuvant chemoimmunotherapy (NACI) or the occurrence of severe immune‐related adverse events (irAEs) may result in the loss of opportunity for radical surgical resection in some patients. Therefore, accurate identification of patients who are most likely to benefit from neoadjuvant immunotherapy is essential, yet predictive markers for selecting potential NACI beneficiaries remain inadequately defined [8].

Compared with tumor tissue analysis, peripheral blood testing offers unique advantages, including convenience, minimal invasiveness, and repeatability, and can comprehensively reflect the systemic immune status and overall tumor burden of patients. By analyzing circulating tumor cells, circulating tumor DNA, immune cell subsets, cytokines, and other potential biomarkers in peripheral blood, numerous studies have sought to identify non‐invasive and clinically applicable indicators associated with NACI response [9]. Routine complete blood count analysis also provides valuable predictive information for immunotherapy efficacy. Among these parameters, the neutrophil‐to‐lymphocyte ratio (NLR) is a simple and effective indicator reflecting the balance between systemic inflammation and immune response. A lower NLR generally indicates better immune function and a less pronounced inflammatory state, which may be associated with improved response to immunotherapy [10].

The NADIM trial further demonstrated that patients achieving pathological complete response (pCR) exhibited significantly higher baseline levels of CD4+ programmed cell death‐1 (PD‐1)+ cells and natural killer cells within peripheral blood mononuclear cells (PBMCs) compared with non‐pCR patients [9]. The T‐cell receptor (TCR), which recognizes tumor antigens presented by the major histocompatibility complex (MHC), exhibits high clonal diversity. Its complementarity determining region 3 (CDR3) serves as the core functional domain for antigen‐specific recognition and is a key determinant of TCR diversity. Characterization and analysis of the TCR repertoire in PBMCs can provide valuable insights into the immune status of circulating antitumor T cells [11]; however, its clinical utility in predicting NACI response in LUSC requires further comprehensive investigation.

Camrelizumab is a high‐affinity humanized monoclonal antibody targeting PD‐1 and has been approved in China for first‐line treatment of advanced NSCLC based on the positive results of the phase III CameL and CameL‐sq trials [12, 13]. However, dedicated clinical evidence regarding the use of camrelizumab in the perioperative setting for resectable locally advanced LUSC remains scarce. Here, we conducted a phase II trial to evaluate the efficacy and safety of perioperative camrelizumab combined with platinum‐based neoadjuvant chemotherapy in patients with resectable stage IIB–IIIB LUSC, and to explore potential peripheral blood biomarkers capable of predicting pathological response to NACI.

## 2 Results

### 2.1 Patient Characteristics

**FIGURE 1:**
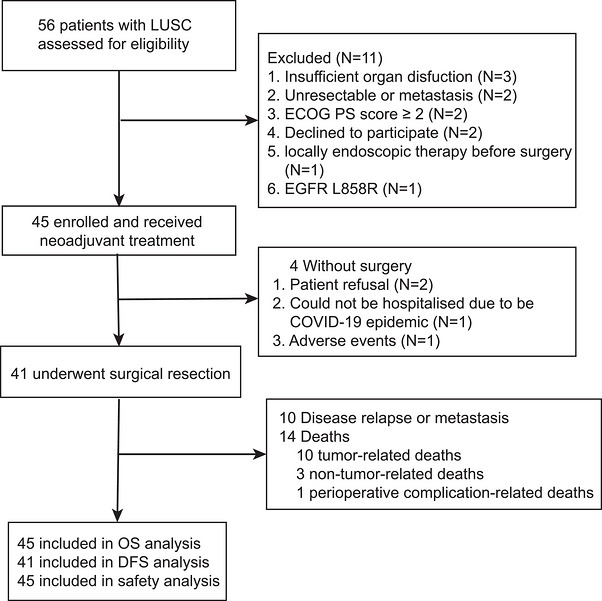
Patient flowchart. COVID‐19, coronavirus disease 2019; DFS, disease‐free survival; ECOG PS, Eastern Cooperative Oncology Group performance status; EGFR, epidermal growth factor receptor; LUSC, lung squamous cell carcinoma; OS, overall survival.

**FIGURE 2:**
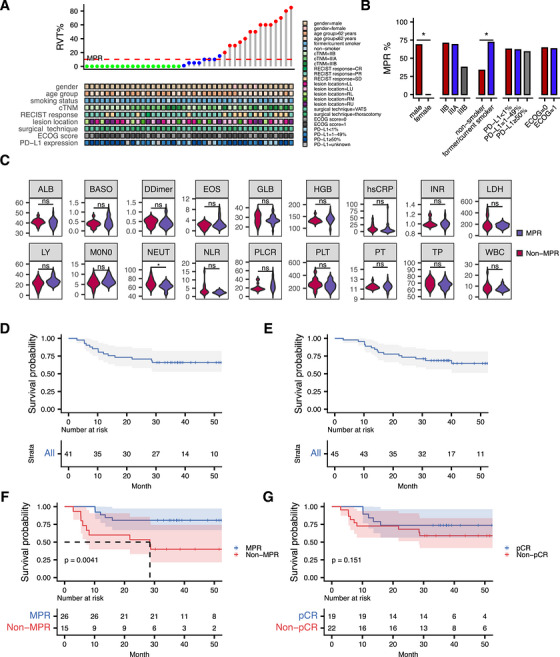
Efficacy analysis and subgroup analyses. (A) Waterfall plot of pathological response to neoadjuvant camrelizumab plus chemotherapy in 41 surgical patients. (B) MPR stratified by clinicopathological variables. *p < 0.05. (C) MPR stratified by parameters derived from blood cell counts, coagulation function, and biochemical tests. *p < 0.05. (D) Kaplan–Meier curve of DFS in 41 surgical patients. (E) Kaplan–Meier curve of OS in all the 45 patients. (F) Kaplan–Meier curves of DFS stratified by MPR. (G) Kaplan–Meier curves of DFS stratified by pCR. ALB, albumin; BASO, percentage of basophils; CR, complete response; cTNM, clinical tumor node metastasis stage; ECOG, Eastern Cooperative Oncology Group; EOS, percentage of eosinophils; GLB, globulin; HGB, hemoglobin; hsCRP, high‐sensitivity C‐reactive protein; INR, international normalized ratio; LDH, lactate dehydrogenase; LL, left lower lobe; LU, left upper lobe; LY, percentage of lymphocytes; MONO, percentage of monocytes; NEUT, percentage of neutrophils; NLR, neutrophil‐to‐lymphocyte ratio; ns, not significant; MPR, major pathological response; pCR, pathological complete response; PD‐L1, programmed cell death‐ligand 1; PLCR, platelet large cell ratio; PLT, platelet count; PR, partial response; PT, prothrombin time; RECIST, Response Evaluation Criteria in Solid Tumors; RL, right lower lobe; RM, right middle lobe; RU, right upper lobe; RVT%, percentage of residual viable tumor cells; SD, stable disease; TP, total protein; VATS, video‐assisted thoracoscopic surgery; WBC, white blood cell count.

Between March 16, 2021, and July 19, 2023, a total of 45 patients were enrolled in this study. The patient flowchart is presented in Figure 1. The median age was 62 years (range, 44–73), and the majority of patients were male (93.3%). Among the 45 patients, 27 (60.0%) had clinical stage III disease. A total of 41 patients (91.1%) underwent PD‐L1 expression testing, among whom 32 (71.1%) were PD‐L1 positive, including 17 (37.8%) with high PD‐L1 expression (Table 1 and Figure 2A).

**TABLE 1:** Baseline characteristics.

Characteristics	Patients (*N* = 45)
Age (years), median (range)	62 (44–73)
Gender, *n* (%)	
Female	3 (6.7)
Male	42 (93.3)
Clinical stage, *n* (%)	
IIB	18 (40.0)
IIIA	19 (42.2)
IIIB	8 (17.8)
ECOG performance status, *n* (%)	
0	15 (33.3)
1	30 (66.7)
Smoking, *n* (%)	
Non‐smoker	10 (22.2)
Former or current smoker	35 (77.8)
PD‐L1 TPS, *n* (%)	
< 1%	9 (20.0)
1%–49%	15 (33.3)
≥ 50%	17 (37.8)
Unknown	4 (8.9)

Abbreviations: ECOG, Eastern Cooperative Oncology Group; PD‐L1, programmed cell death‐ligand 1; TPS, tumor proportion score.

### 2.2 Efficacy

All 45 patients received at least one dose of NACI with camrelizumab and chemotherapy. Due to the coronavirus disease 2019 (COVID‐19) pandemic, one patient was unable to be admitted to the study site according to the study protocol and received treatment at a local hospital; therefore, 44 patients were included in the radiographic response analysis.

Following NACI, two of 44 patients (4.5%) achieved a radiographic complete response (CR), and 29 (65.9%) achieved a partial response (PR), resulting in an objective response rate (ORR) of 70.5%. In the full analysis set, 27 of 45 patients (60.0%; 95% confidence interval [CI]: 45.7%–74.3%) achieved a major pathological response (MPR), including 20 patients (44.4%; 95% CI: 30.2%–60.1%) who achieved pCR. The MPR and pCR rates were 65.9% (27/41) and 48.8% (20/41) in the surgery set (Figure 2B–G and Table S1), respectively.

Among patients with stage IIIB disease, the ORR was 50% (4/8), and the pCR rate was 37.5% (3/8). The median interval between the first NACI dose and surgery was 63 days (range, 37–118). Of the 41 patients who underwent surgery, 16 (39.0%) and 25 (61.0%) underwent lobectomy via video‐assisted thoracoscopic surgery (VATS) and thoracotomy, respectively; two patients (4.8%) initially underwent VATS and were converted to thoracotomy due to hemorrhage. All 41 patients (100%) achieved R0 resection.

As of the data cutoff on October 8, 2025, the median follow‐up duration in the full analysis set was 37.5 months (interquartile range, 24.9–51.1). In the surgical set, 10 patients experienced disease recurrence, including bronchial stump recurrence (*n* = 6) and metastases involving lymph nodes (*n* = 1), brain (*n* = 2), and adrenal gland (*n* = 1). In the full analysis set, 14 deaths occurred, including 10 tumor‐related deaths, one death due to perioperative complications, and three deaths due to non‐tumor‐related causes.

The median disease‐free survival (DFS) and overall survival (OS) were not reached. The 2‐year and 3‐year DFS rates were 70.7% and 65.9%, respectively (Figure 2D). The 2‐year and 3‐year OS rates were 73.3% and 68.7%, respectively (Figure 2E). The MPR group demonstrated significantly longer median DFS compared with the non‐MPR group (*p* = 0.004), while no difference in DFS was observed between the pCR and non‐pCR groups (*p* = 0.15; Figure 2F,G).

### 2.3 Safety

During perioperative therapy, treatment‐related adverse events (TRAEs) occurred in 37 of 45 patients (82.2%). The most common TRAEs were reactive cutaneous capillary endothelial proliferation (RCCEP; 35 [77.8%]), decreased white blood cell count (20 [44.4%]), anorexia (13 [28.8%]), and nausea (11 [24.4%]). Grade 3 or higher TRAEs occurred in nine patients (20.0%), with the most frequent events being decreased white blood cell count (4 [8.9%]) and decreased neutrophil count (3 [6.7%]).

IrAEs occurred in 38 patients (84.4%), including grade ≥ 3 events in five patients (11.1%), which are comparable to rates reported in CheckMate 816 (77% any grade, 0% grade ≥ 3) and KEYNOTE‐671 (74% and 12%) [3, 4]. The most common irAE, regardless of grade, was RCCEP (35 [77.8%]; Table S2).

Discontinuation of camrelizumab occurred in five patients (11.1%). One patient (2.2%) developed grade 3 pneumonia after NACI, resulting in delayed surgery. During the adjuvant phase, four patients (8.9%) experienced interruption of camrelizumab due to grade 4 diarrhea (*n* = 1), grade 3 maculopapular rash (*n* = 1), and grade 3 liver injury (*n* = 2). No irAE‐related deaths occurred, and all events resolved to baseline or grade ≤ 1.

The incidence of grade ≥ 3 TRAEs (20%) and irAE‐related discontinuation (11%) was consistent with ranges reported in previous phase III trials of NACI, including CheckMate 816 (20% grade ≥ 3 TRAEs; 9% discontinuation), KEYNOTE‐671 (24% and 14%), and AEGEAN (27% and 11%) [3, 4, 5]. The clinical courses of representative cases are summarized as follows.

Hepatitis (*n* = 2, grade 3, occurring at adjuvant cycles 4 and 9, respectively) was characterized by alanine aminotransferase (ALT) and aspartate aminotransferase (AST) levels of 480/420 and 395/350 U/L, respectively. Patients were treated with intravenous methylprednisolone at 2 mg/kg/day for 5 days, followed by 1 mg/kg/day for 7 days. Upon reduction of ALT and AST levels to grade ≤ 1, treatment was transitioned to oral prednisone 40 mg/day, which was tapered by 10 mg every 5 days to 10 mg, followed by reduction by 5 mg every 7 days. Camrelizumab was permanently discontinued. Both patients remained disease free at 34 and 41 months, respectively.

Diarrhea (*n* = 1, grade 4, occurring at adjuvant cycle 7) was characterized by nine bowel movements per day. Colonoscopy confirmed immune‐mediated colitis. The patient received intravenous methylprednisolone at 1 mg/kg/day for 5 days, which was reduced to 0.5 mg/kg/day for 5 days after symptom improvement to grade ≤ 1. Subsequently, the patient was transitioned to oral prednisone at 20 mg/day for 1 week, followed by 10 mg/day for 1 week, 5 mg/day for 1 week, and 2.5 mg/day for 1 week, resulting in a total corticosteroid treatment duration of 6 weeks. Camrelizumab was permanently discontinued, and no recurrence was observed.

Pneumonitis (*n* = 1, grade 3, occurring at adjuvant cycle 4): Computed tomography (CT) revealed bilateral ground‐glass opacities. The patient was treated with intravenous methylprednisolone at 2 mg/kg/day for 5 days, followed by 1 mg/kg/day when the radiographic grade was ≤ 1. Subsequently, the patient was switched to oral prednisone at 40 mg/day, which was tapered by 10 mg every 7 days, resulting in a total steroid course of 8 weeks. Pneumonitis resolved completely by week 8. Camrelizumab was permanently discontinued. The patient remained recurrence‐free at 38 months.

Maculopapular rash (*n* = 1, grade 3, occurring at adjuvant cycle 3): Biopsy confirmed lichenoid dermatitis. The patient was treated with topical mometasone furoate cream in combination with oral prednisone at 0.5 mg/kg for 7 days, followed by 20 mg/day tapered by 5 mg every 5 days, resulting in a total treatment duration of 6 weeks. Camrelizumab was permanently discontinued without recurrence.

Thyroiditis (*n* = 1, grade 3, occurring at adjuvant cycle 2): Immune‐mediated thyroiditis presented with biochemical thyrotoxicosis (thyroid‐stimulating hormone [TSH] 0.02 mIU/L; free thyroxine [FT4] 38 pmol/L) but no clinical symptoms. The subsequent hypothyroid phase, characterized by TSH >100 mIU/L, was treated with levothyroxine. Thyroid function stabilized by 8 weeks. Following multidisciplinary team (MDT) discussion, camrelizumab was continued without interruption. The patient remained disease‐free at 46 months.

Surgical complications occurred in 10 (24.4%) of 41 patients, most of which were grade 1 or 2. The most frequent surgical complication was intraoperative bleeding (three cases, 7.3%). One patient died of postoperative lung infection, which was considered unrelated to NACI (Table S2).

### 2.4 Routine Biomarker Analysis

Male patients and former or current smokers exhibited higher MPR rates than female patients and non‐smokers. Comparable MPR rates were observed across patients with different PD‐L1 expression levels (Figure 2B). The percentage of neutrophils (NEUT%) was significantly higher in the non‐MPR group than in the MPR group (Figure 2C).

### 2.5 Exploratory Analysis Between TCR and pCR

**FIGURE 3:**
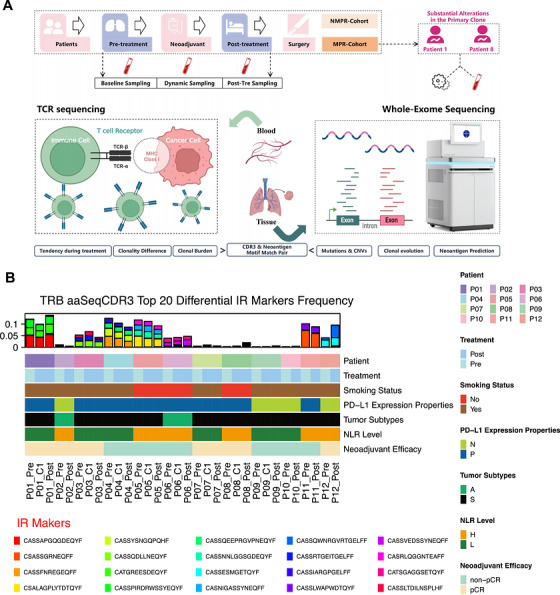
Overview of the exploratory analysis. (A) Flow chart of the specimen collection. (B) Distribution of clinical characteristics and high‐abundance TRB clones in 12 patients. The upper histogram shows the distribution of the top 20 clone abundances, while the markers below indicate the corresponding CDR3 amino acid sequences. H, high; IR, immune repertoire; L, low; MPR, major pathological response; N, negative; NLR, neutrophil‐to‐lymphocyte ratio; NMPR, non‐major pathological response; TCR, T‐cell receptor; TRB, T‐cell receptor beta; P, positive; pCR, pathological complete response; PD‐L1, programmed cell death‐ligand 1.

With patient consent and qualified blood samples, 32 blood samples from 12 patients were successfully collected, of whom six achieved pCR. The design of the exploratory analysis is illustrated in Figure 3A, and the characteristics of these 12 patients are presented in Table S3. One patient received only one cycle of NACI due to worsening respiratory symptoms, whereas the remaining patients received two cycles of NACI.

**FIGURE 4:**
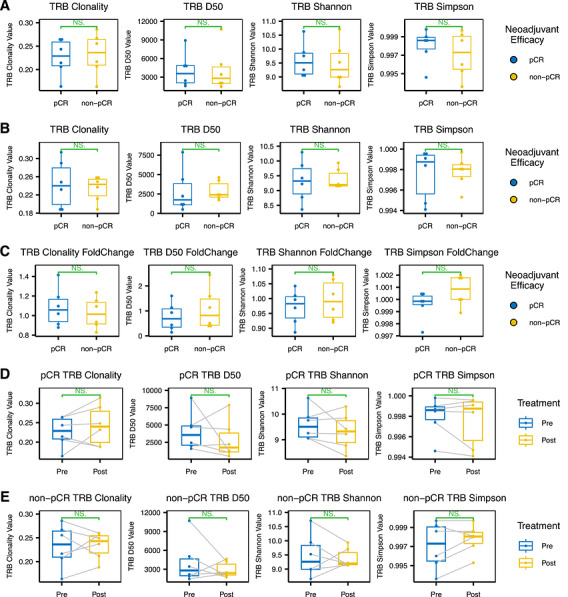
Diversity analysis of TRB in 12 patients. (A) Comparison of TRB diversity before neoadjuvant therapy between pCR and non‐pCR patients. (B) Comparison of TRB diversity after neoadjuvant therapy between pCR and non‐pCR patients. (C) Comparison of fold changes in TRB diversity between pCR and non‐pCR patients. (D) Longitudinal dynamics of TRB diversity indices in patients with pCR. (E) Longitudinal dynamics of TRB diversity indices in patients with non‐pCR. TRB diversity was assessed using clonality index, D50 index, Shannon entropy, and Simpson index, respectively. pCR, pathological complete response; TRB, T‐cell receptor beta.

Saturation curve analysis demonstrated that the TCR sequencing data were reliable and suitable for further analysis (Figure S1). The distribution of high‐abundance TCR clones is shown in Figure 3B. TCR diversity was evaluated using clonality index, D50 index, Shannon entropy, and Simpson index, respectively. All four indices indicated no significant differences between the pCR and non‐pCR groups in the diversity of either TCR alpha (TRA) or TCR beta (TRB) before or after NACI (Figure 4A,B and Figure S2A,B). Furthermore, none of the fold changes in these diversity indices relative to pre‐treatment values after NACI correlated with pCR (Figure 4C and Figure S2C). Dynamic changes in TCR diversity were also assessed at multiple time points before and after NACI. In both the pCR and non‐pCR groups, none of the four indices changed significantly (Figure 4D,E and Figure S2D,E). In addition, samples from nine patients collected after one cycle of NACI showed no correlation between TRB diversity and pCR (Figure S3).

The level of PD‐L1 expression in tumor cells did not correlate with the pre‐treatment diversity of either TRA or TRB (Figure S4). Similarly, the NLR in peripheral blood did not correlate with the pre‐treatment diversity of either TRA or TRB (Figure S5).

We further analyzed the frequencies of the top 100 CDR3 amino acid sequences, the top 50 variable‐joining (V‐J) gene pairs, and the top 10 variable (V) genes. Before and after neoadjuvant therapy, the frequencies of these indicators in both TRA and TRB clones did not differ significantly between the pCR and non‐pCR groups (Figure S6A,B,E,F). In addition, no significant changes were observed in these indicators before and after neoadjuvant therapy within either the pCR or non‐pCR groups (Figure S6C,D,G,H).

### 2.6 Whole‐Exome Sequencing (WES) Analysis

**FIGURE 5:**
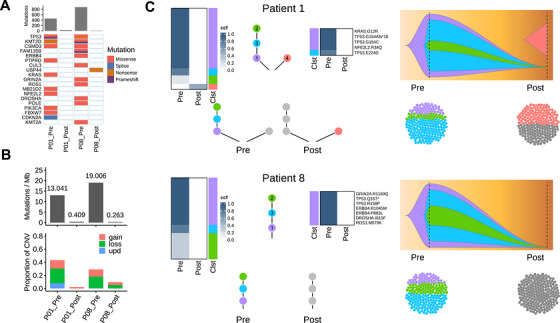
WES analysis of tumor tissue samples from patient #01 (P01) and patient #08 (P08). (A) Distribution of somatic mutations before and after neoadjuvant therapy. (B) Comparison of tumor mutational burden and CNVs before and after neoadjuvant therapy. (C) Clonal analysis before and after neoadjuvant therapy. CNV, copy number variation; WES, whole‐exome sequencing.

Based on available tumor tissues, samples were collected from two patients (patient 1 [P01] and patient 8 [P08]) before and after NACI, both of whom achieved pCR (Table S3). WES was subsequently performed on these samples. Both patients exhibited a marked reduction in molecular variant burden, including single‐nucleotide variants (SNVs) and copy number variations (CNVs), after NACI. Despite achieving pCR, somatic mutations were still detected in both patients after NACI (Figure 5A,B). Clonal analysis showed that all clones present in baseline tumor biopsy samples were completely eliminated after NACI. However, a new clone emerged in the tumor tissue of P01 after NACI, possibly due to tumor heterogeneity (Figure 5C).

### 2.7 Neoantigen Association Analysis

**FIGURE 6:**
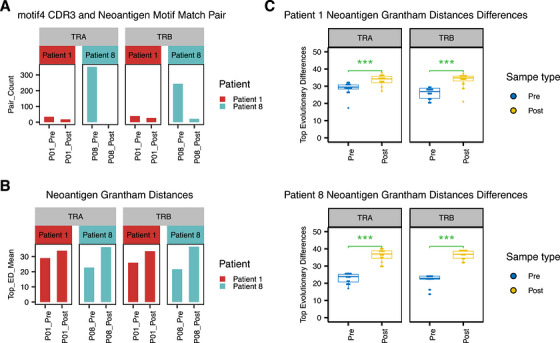
Correlation analysis between TRB and neoantigen motifs in patient #01 (P01) and patient #08 (P08). (A) Number of CDR3 amino acid sequences matching neoantigen motifs before and after neoadjuvant therapy. (B) Mean of the top 20 lowest evolutionary distances between cloned CDR3 amino acid sequences and neoantigen motifs. (C) Intergroup comparison of the lowest top 20 evolutionary distances. TRA, T‐cell receptor alpha; TRB, T‐cell receptor beta.

Correlation analysis between peripheral TCRs and neoantigen motifs was performed in P01 and P08. The number of CDR3 amino acid sequences matching neoantigen‐derived amino acid motifs in blood samples decreased significantly after NACI (Figure 6A), suggesting a reduction in T‐cell binding to tumor antigens following treatment. Consistently, evolutionary analysis using Grantham distance demonstrated a significant increase in the evolutionary distance between CDR3 amino acid sequences and neoantigen motifs after NACI (Figure 6B,C), indicating a substantial decrease in TCR affinity for neoantigens.

**FIGURE 7:**
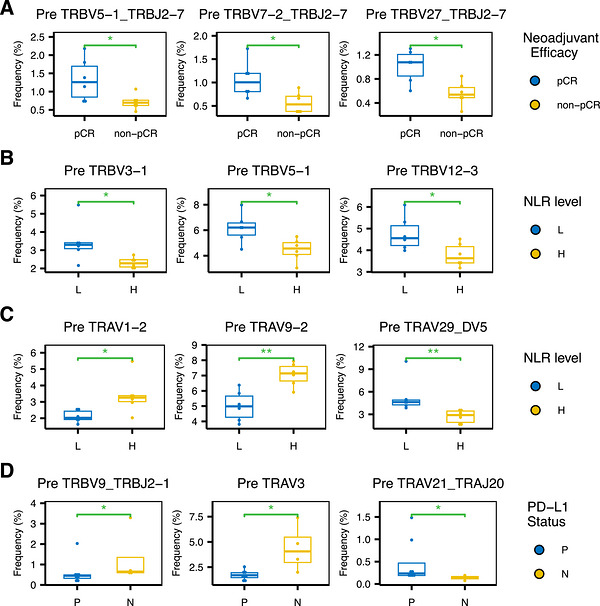
Frequency analysis of specific V–J gene pairs and V genes in TRB and TRA clones from patient #01 (P01) and patient #08 (P08). (A) Comparison between the pCR and non‐pCR groups before neoadjuvant therapy. (B) Comparison of high NLR and low NLR groups before neoadjuvant therapy in TRB clones. (C) Comparison of high NLR and low NLR groups before neoadjuvant therapy in TRA clones. (D) Comparison of PD‐L1‐positive and PD‐L1‐negative groups before neoadjuvant therapy in TRB clones. (E) Comparison of PD‐L1‐positive and PD‐L1‐negative groups before neoadjuvant therapy in TRA clones. H, high; L, low; N, negative; NLR, neutrophil‐to‐lymphocyte ratio; P, positive; pCR, pathological complete response; PD‐L1, programmed cell death ligand 1; TRA, T‐cell receptor alpha; TRB, T‐cell receptor beta.

Finally, we analyzed whether specific CDR3 amino acid sequences, V‐J gene pairs, and V genes differed significantly among groups in pre‐neoadjuvant therapy samples. Due to tumor mutational heterogeneity, no significant differences in CDR3 amino acid sequences were observed across groups. However, significant differences were identified in the frequencies of three V‐J gene pairs in TRB clones between the pCR and non‐pCR groups, whereas no significant differences in V genes or V‐J gene pairs were observed in TRA clones (Figure 7A). Between the high‐NLR and low‐NLR groups, three V genes in TRB clones and three V genes in TRA clones showed significantly different frequencies (Figure 7B,C). Between PD‐L1‐positive and PD‐L1‐negative groups, one V gene pair in TRB clones, as well as one V‐J gene pair and one V gene in TRA clones, exhibited significantly different frequencies (Figure 7D,E).

## 3 Discussion

This phase II study met its primary endpoint of MPR rate with perioperative camrelizumab plus neoadjuvant chemotherapy in patients with resectable stage IIB–IIIB LUSC. Neoadjuvant camrelizumab combined with nab‐paclitaxel and carboplatin achieved MPR and pCR rates of 60.0% and 44.4%, respectively. With a median follow‐up of 37.5 months, the median DFS and OS were not reached. The 3‐year DFS and OS rates were 65.9% and 68.7%, respectively. Treatment‐related toxicity was manageable, and no new safety signals were identified. These findings suggest that this combination may represent a potential neoadjuvant treatment option for LUSC.

In multiple neoadjuvant clinical trials, MPR and pCR have been regarded as surrogate endpoints for survival outcomes. In recent years, a series of phase III trials have confirmed the efficacy of ICIs combined with chemotherapy as neoadjuvant therapy in NSCLC. CheckMate 816 demonstrated that neoadjuvant nivolumab plus chemotherapy significantly increased MPR and pCR rates to 46.8% and 24.0%, respectively, in patients with stage IB–IIIA NSCLC [3]. In KEYNOTE‐671 and RATIONALE‐315, MPR rates reached 30.2% and 56.2%, while pCR rates were 18.1% and 40.7% [4, 14], respectively. In the present study, neoadjuvant camrelizumab plus chemotherapy yielded more favorable pathological responses. These differences may be attributed to variations in the proportion of LUSC, chemotherapy regimens, PD‐1 inhibitors, and patient ethnicity. Additionally, it should be noted that the study population consisted of highly selected surgical candidates following multidisciplinary evaluation.

This phase II trial demonstrated that adding camrelizumab to neoadjuvant chemotherapy for resectable stage IIB–IIIB LUSC is safe and does not compromise treatment delivery or surgical outcomes. Grade ≥ 3 TRAEs occurred in 20% of patients, which is consistent with other immunochemotherapy studies. The most common irAE was RCCEP (77.8%), which was effectively managed with corticosteroids. Other irAEs, including hepatitis, pneumonitis, and thyroiditis, were also observed but were successfully managed without permanent discontinuation of camrelizumab. The favorable safety profile supports the potential of this regimen for further investigation, although validation in larger trials remains necessary.

In this study, biomarker analyses were performed to explore predictors of clinical efficacy of NACI. Correlation analyses were conducted between clinicopathological characteristics, routine blood parameters, and pathological response. Smoking history was associated with a higher MPR rate. Carcinogenic nicotine‐derived nitrosamines in tobacco smoke have been reported to upregulate indoleamine 2,3‐dioxygenase 1 (IDO1) expression through activation of the transcription factor c‐Jun. IDO1 is an enzyme involved in tryptophan (Trp) metabolism, and its upregulation leads to metabolic imbalance, thereby promoting regulatory T‐cell generation and inhibiting CD8+ T‐cell activity [15]. Similar findings have been reported in advanced NSCLC [16, 17]. In China, smoking is strongly associated with male sex. In this study, male patients also exhibited a higher MPR rate. In the non‐MPR group, NEUT% was higher than that in the MPR group. This phenomenon has been frequently reported in immunotherapy studies of malignant tumors, although its underlying mechanism remains unclear [18]. No association was observed between PD‐L1 expression levels and MPR rate. In non‐squamous NSCLC, PD‐L1 expression appears to better predict the benefit of immunotherapy. However, in LUSC, the relationship between PD‐L1 expression and immunotherapy efficacy is less definitive [19]. Therefore, PD‐L1 expression alone may not be an optimal biomarker for evaluating immunotherapy benefit in patients with LUSC, and further studies are required to identify additional predictive biomarkers.

We further explored whether peripheral blood TCR characteristics could serve as biomarkers of response to NACI in patients with locally advanced LUSC. Owing to sample accessibility, dynamic blood samples from 12 patients were obtained. Exploratory TCR analysis identified three V‐J gene pairs in TRB clones that differed significantly between the pCR and non‐pCR groups, suggesting potential response‐associated clonal expansion patterns. However, overall TCR diversity indices (e.g., Shannon entropy and Simpson index) in PBMCs were not associated with pCR, consistent with previous studies indicating that bulk TCR repertoire analyses may obscure tumor‐specific signals [20, 21]. This observation highlights the importance of focusing on tumor antigen‐specific TCR populations rather than global TCR diversity. The observed reduction in tumor neoantigen‐specific TCR clones following NACI in two patients with pCR further supports the hypothesis that effective therapy may reduce tumor‐specific T cell clones due to decreased antigenic burden. These findings are consistent with emerging evidence suggesting that chemotherapy‐induced non‐tumor antigenic alterations may dilute peripheral TCR specificity [22], highlighting the complexity of identifying reliable immunotherapy biomarkers. Future studies with larger cohorts are required to validate these V‐J gene pairs as predictive biomarkers and to elucidate their functional relevance in tumor clearance.

Because peripheral expansion of T‐cell clones can occur between weeks 2 and 4 after initiation of anti‐PD‐1 therapy [23], blood samples were collected before the second cycle of NACI. Previous studies have reported inconsistent findings regarding the association between peripheral blood TCR diversity and clinical outcomes. In a neoadjuvant immunotherapy study in NSCLC, Zhang et al. observed a trend toward higher peripheral TCR diversity in patients with MPR compared with those without MPR at each post‐treatment time point; however, the difference was not statistically significant [23]. The inconsistency among studies may be attributed to multiple factors, including differences in treatment regimens, specific T‐cell subsets, and the assessment of tumor neoantigen‐specific TCRs. Recent findings by Li et al. suggested that baseline TCR diversity was positively associated with durable clinical benefit in patients receiving ICI monotherapy. In contrast, no such association was observed in patients treated with immunotherapy combined with chemotherapy [20]. Additionally, Zhang et al. reported a positive association between intratumoral TCR clonality and MPR in NSCLC patients treated with NACI [23]. Furthermore, higher baseline TCR diversity in peripheral PD‐1+ CD8+ T cells was associated with better responses to ICIs and longer progression‐free survival in patients with NSCLC. However, TCR diversity in unselected PBMCs was not correlated with immunotherapy outcomes [21]. These findings indicate that tumor neoantigen‐specific TCRs, rather than the global TCR repertoire in peripheral blood, may serve as more reliable indicators of clinical outcomes in immunotherapy. The presence of numerous non‐tumor antigen‐specific TCRs may dilute tumor‐specific signals, thereby reducing the overall informativeness of TCR diversity measures with respect to clinical outcomes. Nevertheless, the accurate assessment of tumor neoantigen‐specific T‐cell recognition based solely on TCR sequencing remains challenging. Therefore, WES was performed on tumor tissue samples from two patients who achieved pCR. Consistent with the clinical response, WES revealed a significant reduction in tumor molecular variant burden in both patients following NACI. Neoantigens arise from somatic nonsynonymous mutations in tumor genes and can elicit antigen‐specific T‐cell responses when presented by antigen‐presenting cells [24]. To further investigate the interaction between TCRs and tumor neoantigens, the binding strength and evolutionary distance between CDR3 amino acid sequences and tumor neoantigen sequences were assessed in patients P01 and P08. The number of tumor neoantigens in tissue samples was significantly reduced, reflecting a decreased somatic mutational burden. In these two patients, the number of tumor neoantigen‐specific TCR clones decreased significantly after NACI, whereas overall TCR diversity in PBMCs remained unchanged. In addition, the present study employed a NACI regimen rather than ICI monotherapy. Chemotherapy can induce non‐tumor‐specific mutations, which may generate non‐tumor neoantigen‐specific TCRs [22]. This may partly explain why peripheral blood TCR diversity did not reflect clinical outcomes in patients receiving NACI in the present study. Taken together, these findings suggest that TCR profiling of unselected PBMCs may not accurately reflect the therapeutic efficacy of neoadjuvant immunotherapy combined with chemotherapy in patients with locally advanced LUSC. However, a reduction in tumor neoantigen‐specific TCR clones may be associated with favorable pathological response.

This study has several limitations. The primary endpoint, MPR, is a surrogate endpoint, and it remains unclear whether patients achieving MPR will experience improved long‐term survival, which requires further follow‐up. Survival follow‐up is ongoing and is essential for determining the clinical significance of the treatment strategy. The small sample size from a single center represents another important limitation, which may reduce the robustness of the study conclusions. The absence of randomization and a control group limits the ability to draw definitive conclusions regarding the superiority of the treatment regimen compared with standard therapies. Moreover, the biomarker analyses were exploratory and based on a limited number of pre‐ and post‐treatment samples, indicating that further studies are required for more comprehensive molecular characterization. The lack of paired longitudinal samples further limits the interpretability of the biomarker findings. Finally, PBMCs were not enriched for tumor‐specific T cells, which may have introduced interference from non‐tumor antigen‐specific TCRs. Therefore, these findings should be considered exploratory and require validation in larger NSCLC cohorts.

In conclusion, this study suggests the potential of perioperative camrelizumab combined with neoadjuvant chemotherapy in patients with resectable locally advanced LUSC, demonstrating promising antitumor activity, including in patients with stage IIIB disease. The identification of preoperative blood biomarkers for patient selection warrants further investigation and validation. These findings should be confirmed in future phase III randomized controlled trials.

## 4 Materials and Methods

### 4.1 Study Design and Participants

This single‐arm phase II trial was conducted at Beijing Chest Hospital, China, and was registered at ChiCTR.org.cn (ChiCTR2100044645). The study design is presented in Figure S7. The trial enrolled treatment‐naïve patients aged 18–75 years with histologically confirmed resectable stage IIB–IIIB LUSC (stage IIIB restricted to T3–4N2, according to the eighth edition of the American Joint Committee on Cancer [AJCC] staging system). Surgical resectability was determined by multidisciplinary consultation, including surgeons, radiologists, pathologists, radiation oncologists, and medical oncologists. All patients underwent comprehensive preoperative imaging, including contrast‐enhanced chest and abdominal CT, brain magnetic resonance imaging (MRI), ultrasound of superficial lymph nodes and the abdomen, and bone emission CT (ECT). Positron emission tomography/CT (PET/CT) was also performed, and invasive mediastinal lymph node staging was conducted using endobronchial ultrasound or mediastinoscopy when necessary.

Additional inclusion criteria included an Eastern Cooperative Oncology Group (ECOG) performance status of 0 or 1, at least one measurable target lesion according to Response Evaluation Criteria in Solid Tumors (RECIST) version 1.1 [25], and adequate organ function. Key exclusion criteria included immunodeficiency, tumor invasion of major blood vessels, epidermal growth factor receptor (EGFR) or anaplastic lymphoma kinase (ALK) mutations, chronic hepatitis B virus infection, active infection requiring treatment, a history of malignant tumors within 5 years, and glucocorticoid use within 14 days. Full eligibility criteria are provided in the study protocol.

The trial was conducted in accordance with the Declaration of Helsinki and Good Clinical Practice guidelines and was approved by the Ethics Committee of Beijing Chest Hospital, Capital Medical University (No. YNLX‐2021‐01). Written informed consent was obtained from all participants.

### 4.2 Treatment

Camrelizumab (200 mg) was administered intravenously on Day 1 of each 21‐day cycle for two cycles prior to surgical resection. The neoadjuvant chemotherapy regimen consisted of nab‐paclitaxel (130 mg/m^2^ administered intravenously on Days 1 and 8) and carboplatin (area under the curve [AUC] = 5, administered intravenously on Day 1), given every 3 weeks for two cycles. Surgery was scheduled 4–6 weeks after completion of NACI. After resection, patients received camrelizumab (200 mg intravenously on Day 1 of each 21‐day cycle) for up to 16 cycles or 1 year. CT or contrast‐enhanced CT was performed 30 days after surgery, every 3 months for the first 2 years, every 6 months during Years 3 and 4, and annually thereafter.

### 4.3 Endpoints and Assessments

The primary endpoint was the MPR rate. MPR was defined as the presence of ≤ 10% viable tumor cells in the resected primary tumor specimens [26]. Secondary endpoints included pCR rate (defined as the absence of viable tumor cells in surgical specimens from both the primary tumor and all sampled regional lymph nodes), ORR (defined as the proportion of patients achieving CR or PR according to RECIST v1.1), DFS (defined as the time from surgery to the first occurrence of disease progression, recurrence, or death from any cause), and safety.

Pathological response assessments were independently performed by two senior pathologists. Safety evaluations were conducted every 3 weeks during NACI and every 8 weeks during adjuvant therapy. Adverse events were graded according to the National Cancer Institute Common Terminology Criteria for Adverse Events version 5.0.

### 4.4 Routine Biomarker Measurement

To evaluate organ function, routine peripheral blood tests were performed within 1 week prior to treatment initiation. A series of parameters previously reported as potential predictive indicators were extracted from blood cell counts, coagulation profiles, and biochemical tests, including white blood cell count, hemoglobin, platelet count, percentage of lymphocytes, percentage of monocytes, NEUT%, NLR, percentage of eosinophils, percentage of basophils, platelet large cell ratio, total protein, albumin, globulin, lactate dehydrogenase, high‐sensitivity C‐reactive protein, prothrombin time, international normalized ratio, and D‐dimer.

PD‐L1 expression in tumor cells was assessed by immunohistochemistry using the Dako 22C3 antibody. A total of 100 tumor cells were evaluated under a microscope, and PD‐L1 expression was quantified using the tumor proportion score (TPS). TPS values of <1%, 1%–49%, and ≥ 50% were defined as PD‐L1‐negative, low expression, and high expression, respectively.

### 4.5 TCR Analysis

TCR repertoire libraries were constructed using a 5′ rapid amplification of cDNA ends (5′ RACE) protocol with the SMARTer Human TCR α and β Profiling Kit v2 (Takara Bio). Briefly, total RNA was extracted from PBMCs, followed by first‐strand cDNA synthesis using TCR constant region‐specific primers. A template‐switching oligonucleotide containing unique molecular identifiers (UMIs) was used to capture the 5′ ends of mRNA transcripts. TRA and TRB chains were subsequently amplified by semi‐nested polymerase chain reaction, during which Illumina‐compatible sequencing adapters and unique dual indexes were incorporated. The final libraries were purified, quantified, and sequenced on a NovaSeq 6000 platform using a 2 × 150 bp paired‐end configuration.

Fastp (v0.23.0) was used for quality control. Using MiXCR (v3.0.13), UMI‐corrected reads were aligned to V, D, and J gene segments from the IMGT database [27, 28]. Gene usage, rearrangement patterns, and CDR3 sequences were obtained through read assembly and annotation. TCR diversity was evaluated using clonality index, D50 index, Shannon entropy, and Simpson index, respectively. Fisher's exact test was used to compare the numbers of contracted and expanded clones during treatment.

### 4.6 WES Sequencing and Analysis

Genomic DNA was extracted from blood samples and fragmented using NEBNext DNA Fragmentase (NEB). Target enrichment was performed using the SureSelect Human All Exon V6 Kit (G9705A‐M, Agilent Technologies). Sequencing was conducted on an Illumina NovaSeq 6000 platform (San Diego). High‐quality reads were retained using Illumina filter pipelines for downstream analyses. All WES procedures and reagent support were provided by Beijing Novogene Co., Ltd.

Clean reads were aligned to the human reference genome (UCSC hg19) using Burrows‐Wheeler Aligner (BWA v0.7.17). Picard tools (v2.18.9) were used to merge BAM (Binary Alignment/Map) files from the same patient regions. SAMtools (v1.8) was used for statistical processing of BAM files. Somatic SNVs and insertions and deletions (InDels) were identified using VarScan2 and MuTect2 with default parameters [20]. All variants were annotated using ANNOVAR (December 14, 2015) [29]. Somatic CNVs were evaluated by comparing absolute copy number profiles using ABSOLUTE, CNVKit, ASCAT, and Battenberg.

### 4.7 Phylogenetic Analysis

ASCAT was used to estimate the cancer cell fraction (CCF), and the “nmut” value for each mutation was calculated. The “nmut” value was derived from CCF multiplied by the corresponding copy number, where variant allele frequency (VAF), tumor purity, tumor locus‐specific copy number (CNt), and normal locus‐specific copy number (CNn; CNn = 2) were incorporated. Subsequently, the expected mutation copy number was calculated based on VAF, and mutations were assigned to local integer copy number states using a maximum likelihood approach. For each mutation, observed variant counts were used with a reference defined as VAF = 0.5 × preclustering CCF, major allele copy number = 2, minor allele copy number = 0, and purity = 0.5. Clonal and subclonal mutations were grouped based on preclustering CCF.

PyClone was run with 10,000 iterations, burn‐in = 1000, var_prior = “BB,” and ref_prior = “normal.” Clonal deconvolution results from PyClone were used as input for phylogenetic tree construction in CITUP (v0.1.1; https://github.com/amcpherson/citup) [30], with default parameters for QIP mode, mutation clusters, and mean CCF. Mutation clusters were filtered, and trees were ranked using the Bayesian information criterion (BIC), with the optimal solution selected as the tree with the minimum BIC. The Clonevol package was used to characterize subclonal architecture in each tumor region, and phylogenetic trees were generated using Graph software.

### 4.8 Neoantigen Prediction

Human leukocyte antigen (HLA) class I genotypes were inferred from matched normal BAM files using POLYSOLVER (v1.0) with default parameters. Briefly, sequencing reads from WES data mapping to HLA loci were extracted and aligned to IMGT reference alleles using Novoalign within the POLYSOLVER framework. Somatic variants identified by VarScan2 and Mutect2 were merged and annotated using the Ensembl Variant Effect Predictor (VEP, v84) with the “downstream” and “wild‐type” plugins [31]. Nonsynonymous SNVs and InDels were retained for neoantigen prediction. High‐confidence neoantigen candidates, defined as 8‐ to 11‐mer peptides with predicted MHC binding affinities < 500 nM, were prioritized using the pVACseq (v4.0.9) pipeline. Epitope binding was modeled using NetMHC and NetMHCpan algorithms, followed by stringent filtering based on sequencing depth (tumor ≥ 10×, normal ≥ 5×) and allelic fraction thresholds (tumor VAF ≥ 10%, normal VAF ≤ 2%). To identify immunogenic variants, the binding affinity fold change (ratio of wild‐type to mutant IC50) was calculated. Candidates with a fold change > 2, indicating a significant increase in binding stability relative to the wild‐type peptide, were defined as high‐priority neoantigens for downstream analysis.

### 4.9 Antigen Motif Matching

CDR3 amino acid sequences (CDR3 clone typing) were obtained for each patient sample based on TCR sequencing data. Public databases (VDJdb and McPAS‐TCR) were used to retrieve matched datasets, including amino acid sequences, V gene, J gene, and HLA typing information, and analyses were performed separately for TRA and TRB chains [32, 33]. Using epitope sequences extracted from open‐access databases as background, core epitope‐related motifs were identified. Corresponding WES data were integrated, HLA typing information was obtained, and neoantigen mutant epitope sequences (MT‐epitopes) were identified. A k‐mer analysis (based on response fold change and best MT score) and motif mining were performed to identify neoantigen epitope motifs derived from WES data. The number of matches between WES‐derived neoantigen epitope motifs and core epitope‐related motifs was calculated. Motif analysis was performed using the R package Immunarch (v0.4.3).

### 4.10 Affinity Between CDR3 and Amino Acid Sequences of Novel Antigens

Based on the calculation framework for HLA evolutionary divergence, the affinity between CDR3 amino acid sequences and novel WES‐derived antigen sequences was calculated [34]. Briefly, CDR3 amino acid sequences and tumor WES‐derived neoantigen amino acid sequences were normalized within the TCR immune repertoire dataset, and two FASTA files of equal length were constructed. The Grantham distance matrix for each amino acid substitution was calculated, and the average of the 20 lowest evolutionary distances was used to represent binding strength.

### 4.11 Statistical Analysis

The expected MPR rate for patients with locally advanced LUSC receiving neoadjuvant camrelizumab plus chemotherapy was assumed to be 40%, compared with a historical MPR rate of 20% [35]. If the lower bound of the 95% CI for the MPR rate exceeded 20%, the regimen was considered promising. A sample size of 36 patients was calculated to provide at least 80% power to reject the null hypothesis at a one‐sided alpha level of 5%. To account for a 25% dropout rate due to the impact of the COVID‐19 pandemic, a total of 45 patients was required.

Efficacy analyses were performed in the full analysis set (patients receiving at least one dose of NACI) and the surgery set (patients receiving at least one dose of NACI and undergoing surgery). Safety analyses were conducted in the full analysis set. Continuous variables are presented as median (range or interquartile range) and were compared using Student's *t*‐test or the Mann–Whitney *U* test, as appropriate. Categorical variables are presented as frequencies (percentages) and were compared using Fisher's exact test or the chi‐squared test, as appropriate. The 95% CIs for the MPR and pCR rates were estimated using the Clopper–Pearson method. DFS and OS were estimated using the Kaplan–Meier method and compared using the log‐rank test. A *p* value < 0.05 was considered statistically significant. Statistical analyses and visualization were performed using R (version 4.2.0) and GraphPad Prism (version 8.0).

## Author Contributions

M.H., C.W., and T.Z. conceived and designed the study. All authors collected the data. All authors analyzed and interpreted the data. M.H., C.W., and T.Z. wrote the initial draft of the manuscript. All authors contributed to critical revision of the manuscript for important intellectual content. T.Z. provided administrative, technical, and material support and supervised the study. All authors approved the final version of the manuscript for submission.

## Funding

This study was supported by the Beijing Municipal Administration of Hospitals Incubating Program and Beijing Municipal Science and Technology Commission (No. PX2024058), the Tongzhou Technology Project (No. KJ2024CX041), and the Beijing Municipal Public Welfare Development and Reform Pilot Project for Medical Research Institutes (No. JYY2023‐14 and JYY2023‐15). The authors thank all patients and their families for participation in this trial.

## Ethics Statement

The trial (ChiCTR2100044645) was conducted in accordance with the Declaration of Helsinki and Good Clinical Practice guidelines and was approved by the Ethics Committee of Beijing Chest Hospital, Capital Medical University (No. YNLX‐2021‐01). All participants provided written informed consent.

## Conflicts of Interest

The authors declare that no conflicts of interest.

## Supporting information




**Supporting Information**: mco270793‐sup‐0001‐SuppMat.pdf

## Data Availability

Raw sequencing data generated in this study are available in the Genome Sequence Archive for Humans (GSA‐Human; https://ngdc.cncb.ac.cn/gsa/) under accession number HRA017280, subject to approved access. Other datasets generated during the current study are available from the corresponding author upon reasonable request. The study protocol is provided with the article.
