# Supplementary material for: A Phase II Trial of Perioperative Camrelizumab Plus Neoadjuvant Chemotherapy in Resectable Stage IIB–IIIB Lung Squamous Cell Carcinoma
Source: MedComm (2020). 2026 Jun 15;7(7):e70793. doi: 10.1002/mco2.70793 (PMC13269839; doi:10.1002/mco2.70793)
Supplement: Supplementary file 1 — Supporting Information: mco270793‐sup‐0001‐SuppMat.pdf [file MCO2-7-e70793-s001.pdf]

A Phase II Trial of Perioperative Camrelizumab Plus Neoadjuvant Chemotherapy in Resectable Stage IIB–IIIB Lung Squamous Cell Carcinoma

Mingming Hu[1,2], Shuku Liu[3], Yi Han[3], Feng Wang[3], Yang Liu[3], Baohua Lu[1,2], Hongxia Li[1,2], Yuan Gao[1,2], Ying Wang[1,2], Wei Yang[3], Kang Shi[3], Bo Xiao[3], Qunhui Wang[3], Juan Du[4], Haifeng Lin[4], Xiaomi Li[1], Chong Wang[3,*], Tongmei Zhang[1,2,*]

[1]Department of Oncology, Beijing Chest Hospital, Capital Medical University, Beijing, 101149, China
[2]Laboratory for Clinical Medicine, Capital Medical University, Beijing, 100069, China
[3]Department of Thoracic Surgery, Beijing Chest Hospital, Capital Medical University, Beijing, 101149, China
[4]Department of Pathology, Beijing Chest Hospital, Capital Medical University, Beijing, 101149, China

***Corresponding authors**
Tongmei Zhang, Department of Oncology, Beijing Chest Hospital, Capital Medical University, Beijing, 101149, China
E-mail: tongmeibj@163.com
Chong Wang, Department of Thoracic Surgery, Beijing Chest Hospital, Capital Medical University, Beijing, 101149, China
E-mail: wangchong.pku@foxmail.com

Captions for Supplementary Materials

Table S1. Tumor response

Table S2. Adverse events

Table S3. Characteristics of patients in the exploratory analysis

Figure S1. Saturation curve analysis of T cell receptor sequencing.

Figure S2. Diversity analysis of T cell receptor alpha (TRA).

Figure S3. Diversity analysis of T cell receptor beta (TRB) in nine patients after the first cycle of treatment.

Figure S4. Correlations between T cell receptor diversity and the level of programmed cell death-ligand 1 (PD-L1) expression in tumor cells before neoadjuvant therapy.

Figure S5. Correlations between T cell receptor diversity and the neutrophil-to-lymphocyte ratio (NLR) before neoadjuvant therapy.

Figure S6. Frequency analysis of the top 100 CDR3 amino acid sequences, the top 50 V-J gene pairs, and the top 10 V genes in T cell receptor alpha (TRA) clones and T cell receptor beta (TRB) clones.

Figure S7. Trial design.

Table S1. Tumor response

Response	Surgery set	Full analysis set
Pathological response, n (%)	n=41	n=45
Major pathological response	27 (65.9)	27 (60.0)
Pathological complete response	20 (48.8)	20 (44.4)
Radiographic response, n (%)	n=41	n=44*
Complete response	1 (2.4)	2 (4.5)
Partial response	27 (65.9)	29 (65.9)
Stable disease	13 (31.7)	13 (29.5)
Progressive disease	0	0
Objective response	28 (68.3)	31 (70.5)

*Due to the coronavirus disease 2019 pandemic, one patient was unable to be admitted to the study site according to the study protocol and received treatment at a local hospital; therefore, 44 patients were included in the radiographic response analysis.

Table S2. Adverse events

Events	Patients, n (%)	
	Any grade	Grade ≥3
**Treatment-related adverse events (n=45)**		
Reactive cutaneous capillary endothelial proliferation	35 (77.8)	0
White blood cell count decreased	20 (44.4)	4 (8.9)
Anorexia	13 (28.8)	0
Nausea	11 (24.4)	0
Neutrophil count decreased	7 (15.5)	3 (6.7)
Anemia	6 (13.3)	0
Thyroiditis	9 (20.0)	1 (2.2)
Platelet count decreased	3 (6.7)	0
Pneumonia	2 (4.4)	1 (2.2)
Vomiting	2 (4.4)	0
ALT/AST increased	4 (8.8)	1 (2.2)
Maculopapular rash	1 (2.2)	1 (2.2)
Diarrhea	1 (2.2)	1 (2.2)
**Immune-related adverse events (n=45)**		
Reactive cutaneous capillary endothelial proliferation	35 (77.8)	0
Pneumonia	2 (4.4)	1 (2.2)
Thyroiditis	9 (20.0)	1(2.2)
Maculopapular rash	1 (2.2)	1 (2.2)
Diarrhea	1 (2.2)	1 (2.2)
ALT/AST increased	3 (6.7)	2 (4.4)
**Surgical complications (n=41)**		
Intraoperative bleeding	3 (7.3)	0
Lung infection	2 (4.9)	1 (2.4)
Pleural effusion	2 (4.9)	0
Postoperative pneumothorax	1 (2.4)	0
Perioperative death	1 (2.4)	1 (2.4)
Postoperative atelectasis	1 (2.4)	0
Bronchopleural fistula	1 (2.4)	0

ALT, alanine aminotransferase; AST, aspartate aminotransferase.

Table S3. Characteristics of patients in the exploratory analysis

Patient ID	Gender	Age (years)	Smoking status	PD-L1 TPS	Baseline NLR	Pathological response	RVT (%)	TNM stage	Surgical method	NACI cycles	Sampling points
1	Male	65	Yes	80%	Low (1.92)	pCR	0	IIIA	VATS	2	Pre-NACI, C1, Post-NACI
2	Male	71	Yes	0%	High (3.79)	pCR	0	IIIA	VATS	2	Pre-NACI, Post-NACI
3	Male	51	Yes	2%	Low (2.38)	pCR	0	IIIA	VATS	2	Pre-NACI, C1, Post-NACI
4	Male	72	Yes	5%	Low (2.08)	Non-pCR	15	IIB	Thoracotomy	2	Pre-NACI, C1, Post-NACI
5	Male	59	No	90%	High (3.34)	Non-pCR	70	IIIA	Thoracotomy	2	Pre-NACI, C1, Post-NACI
6	Female	59	No	30%	High (2.78)	Non-pCR	50	IIIB	VATS	2	Pre-NACI, C1, Post-NACI
7	Male	69	Yes	45%	Low (1.64)	pCR	0	IIIA	Thoracotomy	2	Pre-NACI, C1, Post-NACI
8	Male	63	No	10%	High (3.21)	pCR	0	IIIB	VATS	2	Pre-NACI, C1, Post-NACI
9	Male	62	Yes	0%	Low (2.38)	Non-pCR	60	IIIA	Thoracotomy	2	Pre-NACI, C1, Post-NACI
10	Male	58	Yes	0%	Low (1.74)	Non-pCR	60	IIIB	Thoracotomy	2	Pre-NACI, Post-NACI
11	Male	73	Yes	0%	High (7.33)	Non-pCR	20	IIIB	Thoracotomy	1	Pre-NACI, Post-NACI (=C1)
12	Male	54	Yes	1%	High (3.89)	pCR	0	IIIA	VATS	2	Pre-NACI, Post-NACI

PD-L1, programmed cell death-ligand 1; TPS, tumor proportion score; NLR, neutrophil-to-lymphocyte ratio; pCR, pathological complete response; RVT, residual viable tumor; NACI, neoadjuvant chemoimmunotherapy; VATS, video-assisted thoracoscopic surgery; Pre-NACI, before neoadjuvant therapy; C1, after the first cycle of neoadjuvant therapy; Post-NACI, after the completion of neoadjuvant therapy.

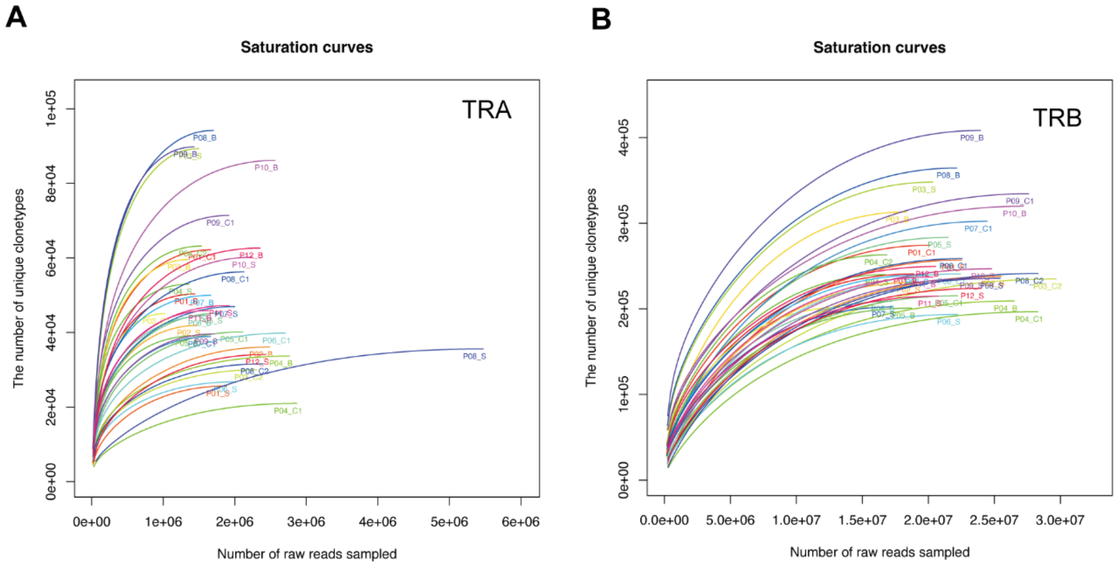

Figure S1. Saturation curve analysis of T cell receptor sequencing. A, T cell receptor alpha (TRA). B, T cell receptor beta (TRB).

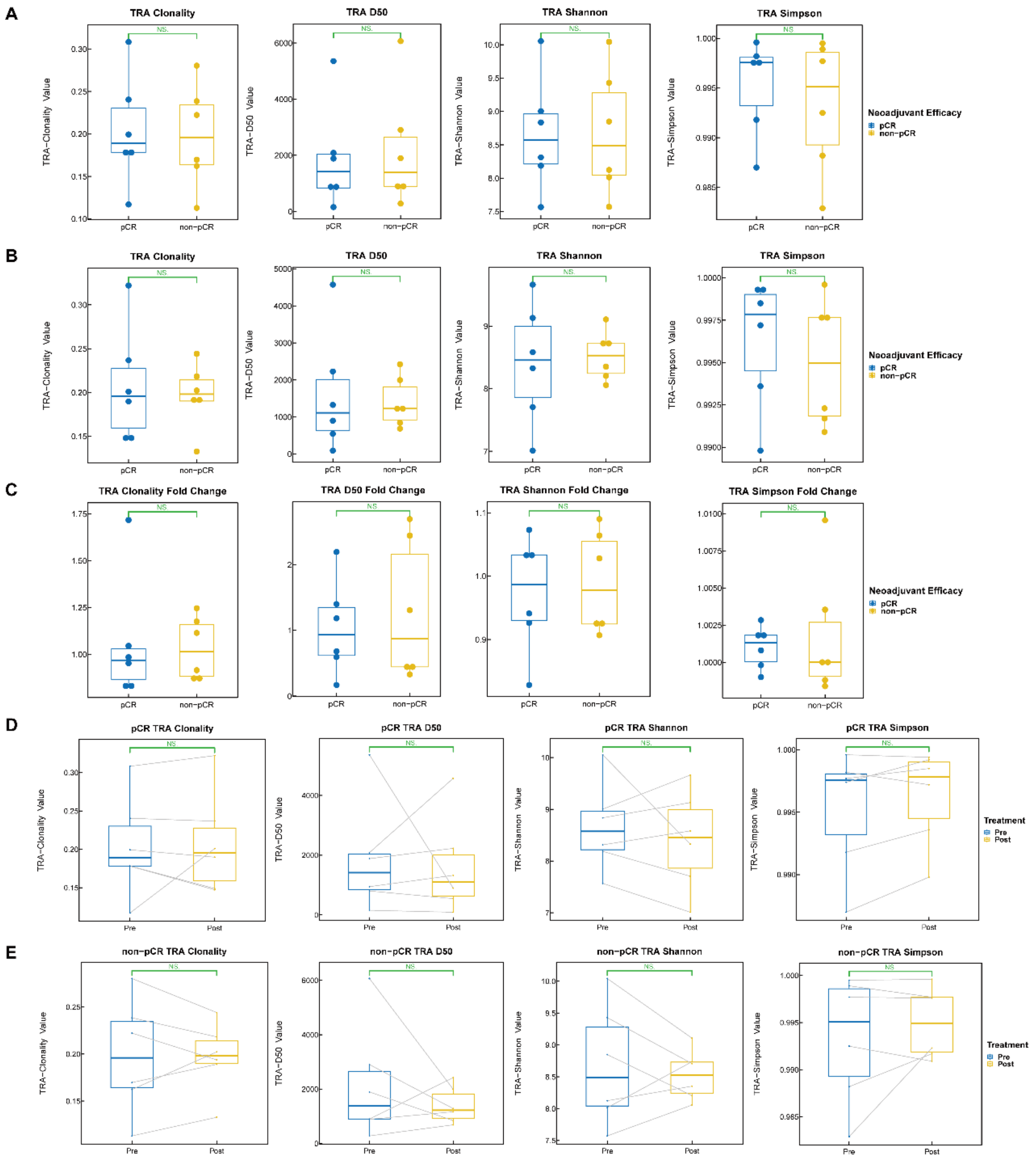

Figure S2. Diversity analysis of T cell receptor alpha (TRA). TRA diversity was assessed using Shannon's entropy, Simpson's index, D50 index, and clonality index. A, Comparison of TRA diversity before neoadjuvant therapy between patients with pathological complete response (pCR) and those with non-pCR. B, Comparison of TRA diversity after neoadjuvant therapy between pCR and non-pCR patients. C, Comparison of fold changes in TRA diversity between pCR and non-pCR patients. D, Dynamics of TRA diversity indices in patients with pCR. E, Dynamics of TRA diversity indices in patients with non-pCR.

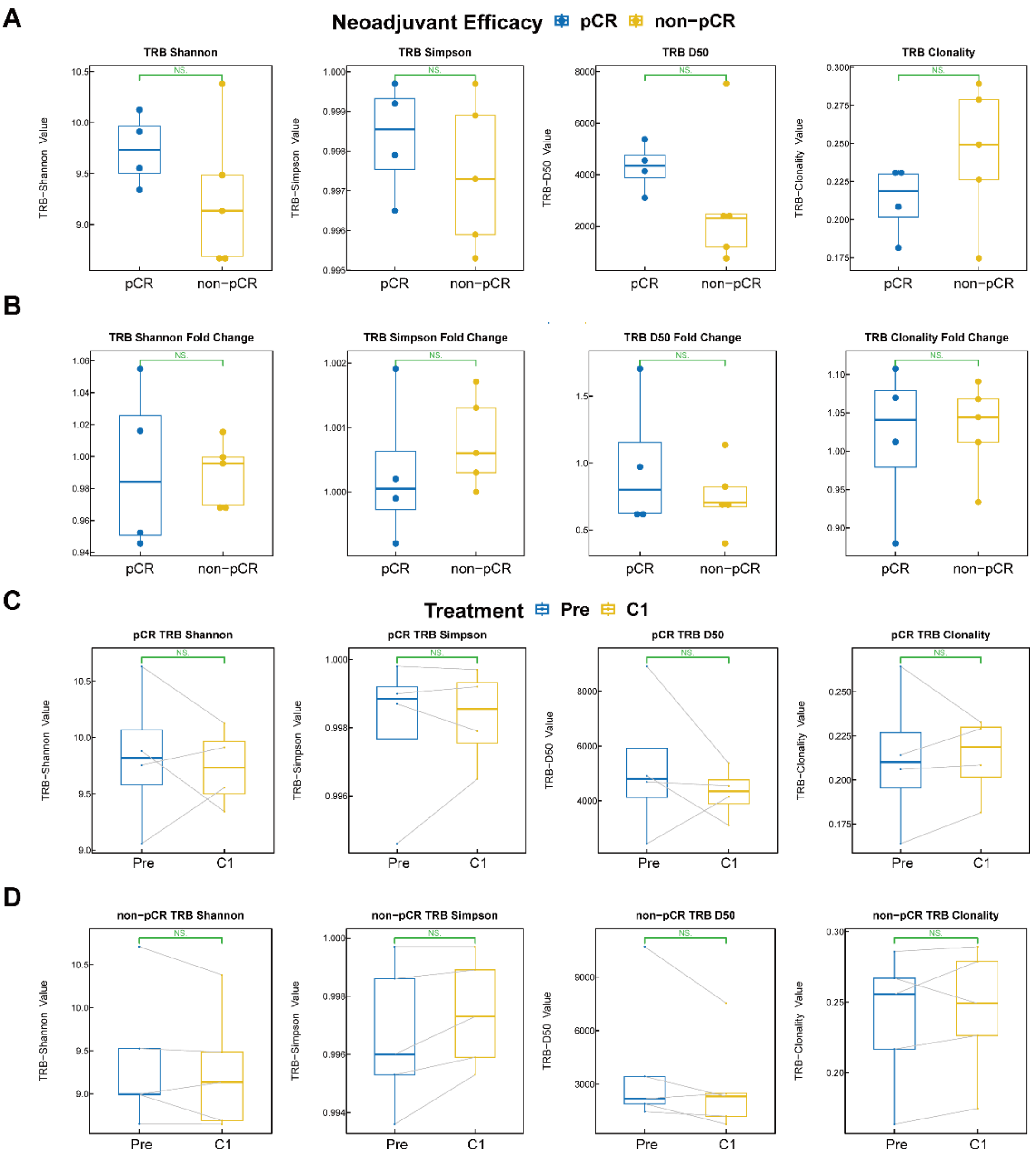

Figure S3. Diversity analysis of T cell receptor beta (TRB) in nine patients after the first cycle of treatment. A, Comparison of TRB diversity before neoadjuvant therapy between patients with pathological complete response (pCR) and those with non-pCR. B, Comparison of TRB diversity after one cycle of neoadjuvant therapy between pCR and non-pCR patients. C, Dynamics of TRB diversity indices in patients with pCR during the first cycle of treatment. D, Dynamics of TRB diversity indices in patients with non-pCR during the first cycle of treatment. C1, the first cycle of treatment.

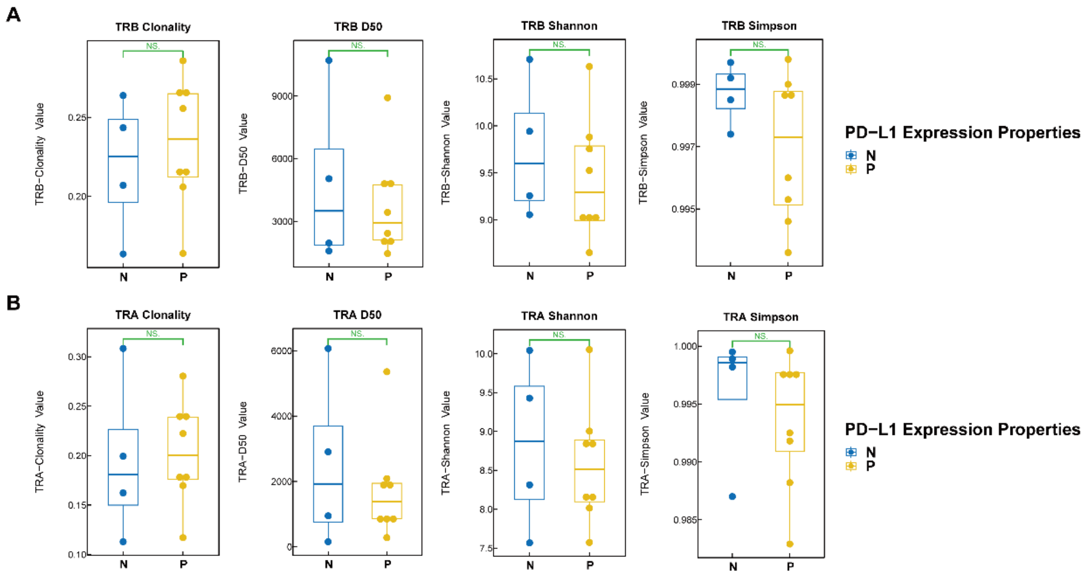

Figure S4. Correlations between T cell receptor diversity and the level of programmed cell death-ligand 1 (PD-L1) expression in tumor cells before neoadjuvant therapy. A, T cell receptor beta (TRB) diversity. B, T cell receptor alpha (TRA) diversity. N, negative; P, positive.

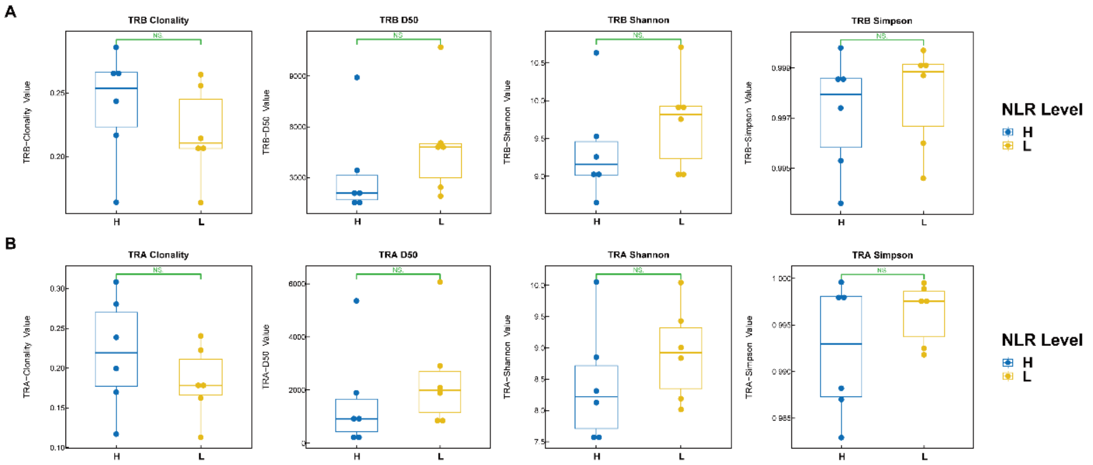

Figure S5. Correlations between T cell receptor diversity and the neutrophil-to-lymphocyte ratio (NLR) before neoadjuvant therapy. A, T cell receptor beta (TRB) diversity. B, T cell receptor alpha (TRA) diversity. H, high; L, low.

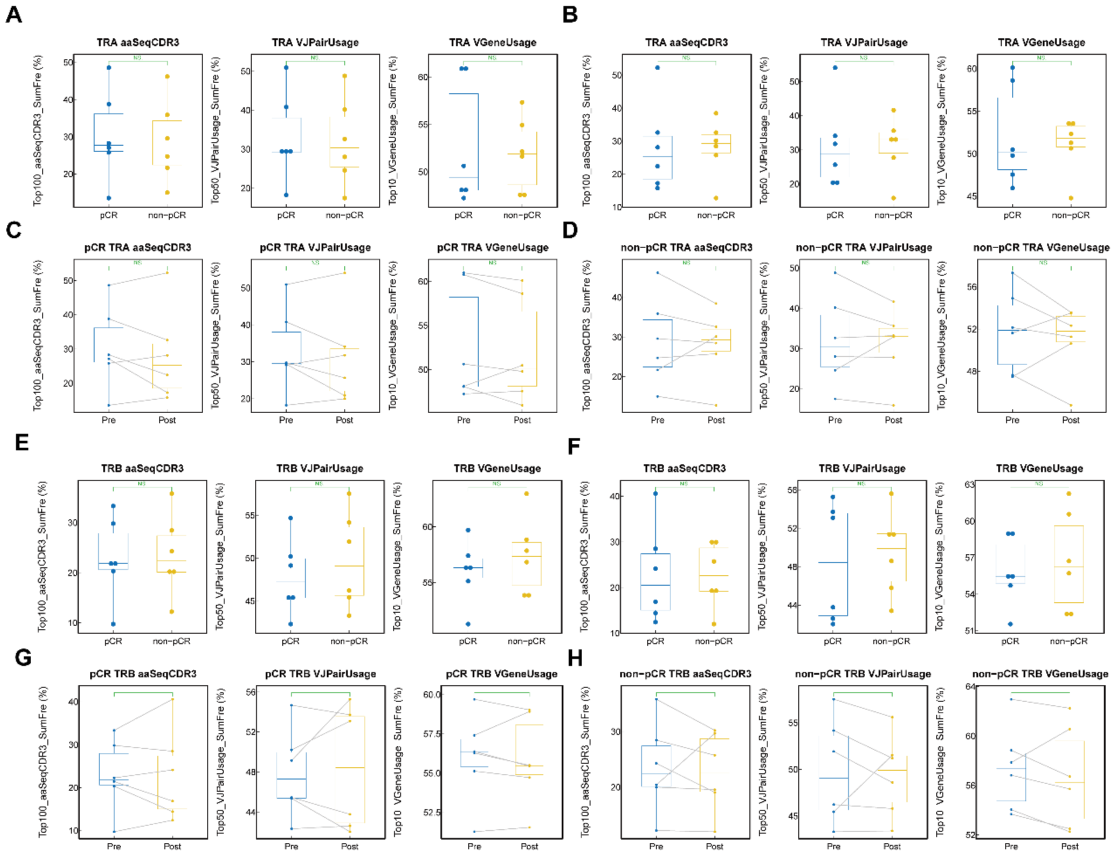

Figure S6. Frequency analysis of the top 100 CDR3 amino acid sequences, the top 50 V-J gene pairs, and the top 10 V genes in T cell receptor alpha (TRA) clones and T cell receptor beta (TRB) clones. A, Comparison of the pathological complete response (pCR) and non-pCR groups before neoadjuvant therapy in TRA clones. B, Comparison of the pCR and non-pCR groups after neoadjuvant therapy in TRA clones. C, Comparison before and after neoadjuvant therapy in the pCR group in TRA clones. D, Comparison before and after neoadjuvant therapy in the non-pCR group in TRA clones. E, Comparison of the pCR and non-pCR groups before neoadjuvant therapy in TRB clones. F, Comparison of the pCR and non-pCR groups after neoadjuvant therapy in TRB clones. G, Comparison before and after neoadjuvant therapy in the pCR group in TRB clones. H, Comparison before and after neoadjuvant therapy in the non-pCR group in TRB clones.

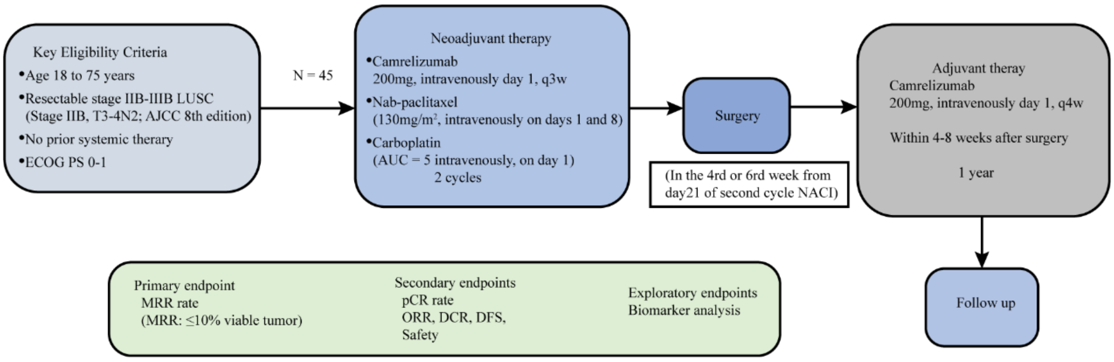

Figure S7. Trial design. LUSC, lung squamous cell carcinoma; AJCC, American Joint Committee on Cancer; ECOG PS, Eastern Cooperative Oncology Group performance status; AUC, area under curve; NACI, neoadjuvant chemoimmunotherapy; MPR, major pathological response; pCR, pathological complete response; ORR, objective response rate; DCR, disease control rate; DFS, disease-free survival.
